# Estimating species – area relationships by modeling abundance and frequency subject to incomplete sampling

**DOI:** 10.1002/ece3.2244

**Published:** 2016-06-17

**Authors:** Yuichi Yamaura, Edward F. Connor, J. Andrew Royle, Katsuo Itoh, Kiyoshi Sato, Hisatomo Taki, Yoshio Mishima

**Affiliations:** ^1^ Graduate School of Agriculture Hokkaido University Nishi 9, Kita 9 Kitaku Sapporo Hokkaido 060‐8589 Japan; ^2^ Department of Forest Vegetation Forestry and Forest Products Research Institute 1 Matsunosato Tsukuba Ibaraki 305‐8687 Japan; ^3^ Department of Biology San Francisco State University 1600 Holloway Avenue San Francisco California 94132; ^4^ Patuxent Wildlife Research Center U. S. Geological Survey Laurel Maryland 20708; ^5^ Itoh Research of Applied Plant Studies Katsura‐machi 560‐114 Satsunai Makubetsu Nakagawa County Hokkaido 089‐0545 Japan; ^6^ Department of Forest Entomology Forestry and Forest Products Research Institute 1 Matsunosato Tsukuba Ibaraki 305‐8687 Japan; ^7^ National Institute for Environmental Studies 16‐2 Onogawa Tsukuba Ibaraki 305‐8506 Japan

**Keywords:** Species‐area relationship, density–area relationship, imperfect detection, incomplete spatial coverage, null model, random placement model, species richness estimator

## Abstract

Models and data used to describe species–area relationships confound sampling with ecological process as they fail to acknowledge that estimates of species richness arise due to sampling. This compromises our ability to make ecological inferences from and about species–area relationships. We develop and illustrate hierarchical community models of abundance and frequency to estimate species richness. The models we propose separate sampling from ecological processes by explicitly accounting for the fact that sampled patches are seldom completely covered by sampling plots and that individuals present in the sampling plots are imperfectly detected. We propose a multispecies abundance model in which community assembly is treated as the summation of an ensemble of species‐level Poisson processes and estimate patch‐level species richness as a derived parameter. We use sampling process models appropriate for specific survey methods. We propose a multispecies frequency model that treats the number of plots in which a species occurs as a binomial process. We illustrate these models using data collected in surveys of early‐successional bird species and plants in young forest plantation patches. Results indicate that only mature forest plant species deviated from the constant density hypothesis, but the null model suggested that the deviations were too small to alter the form of species–area relationships. Nevertheless, results from simulations clearly show that the aggregate pattern of individual species density–area relationships and occurrence probability–area relationships can alter the form of species–area relationships. The plant community model estimated that only half of the species present in the regional species pool were encountered during the survey. The modeling framework we propose explicitly accounts for sampling processes so that ecological processes can be examined free of sampling artefacts. Our modeling approach is extensible and could be applied to a variety of study designs and allows the inclusion of additional environmental covariates.

## Introduction

The observation that species richness increases with area, the species–area relationship (SAR), is one of the few general laws in ecology (Lawton 1999). Many hypotheses have been invoked to mechanistically explain this ubiquitous pattern (Connor and McCoy 1979; Triantis et al. 2012). Explanations commonly focus on how individuals within a community are apportioned into species, and how the abundance of individual species scales with area (Arrhenius 1921; Preston 1962; MacArthur and Wilson 1967; May 1975; Coleman et al. 1982). These hypotheses deal with habitat patches or island systems and assume that population densities of individual species remain constant irrespective of area (“constant density hypothesis”).

Although most of the research on SARs has focused on the functional form of SARs (Connor and McCoy 1979; Triantis et al. 2012), how to collect the data and estimate species richness to generate SARs has received little attention. While Scheiner (2003) provides a classification of several types of SARs, we focus here primarily on SARs that involve independent units, Type IV SARs (Scheiner 2003). Classically, the data used for independent SARs have amounted to no more than lists of species found at a number of sites such as true islands, or geographic or political units. In most instances, no specific sampling design is discussed, and these lists often represent the combined work of numerous naturalists who visit, collect, and describe the species found on each site over many years. The actual sampling effort devoted to produce a species list, which is likely to be greater when the area studied is larger, is unknown, and the “net” probability of detecting a species (i.e., detection probability of at least one individual) is expected to be a function of sampling effort and possibly area. On the other hand, studies of habitat patches use specific sampling designs in which sampling effort may be held constant for all sized patches or may increase for larger patches, but not necessarily in direct proportion to patch area. For example, Lynch and Whigham (1984) used point counts to estimate species richness of birds in forest fragments with one point used in patches <50 ha, two points in patches between 50 and 100 ha, and three points for patches above 100 ha, even though their largest patches exceed 1000 ha. While the authors described their sampling design, they did not use information from the sampling design to adjust estimates of species richness for differences in sampling effort among patches. Therefore, based on current and past sampling practices, SARs may be confounded with sampling effort (Cam et al. 2002) leading to the misestimation of the relationship between species richness and area. For example, Connor and Simberloff (1978) found that the best predictor of plant species richness in the Galapagos Islands was the number of botanical collecting trips to each island, rather than island area. This illustrates that sampling effort is an important determinant of observed species richness and is usually confounded with island or patch area. Cam et al. (2002) recommended that future studies of SARs explicitly account for sampling processes so that ecological processes could be examined free of sampling artefacts.

**Figure 1:**
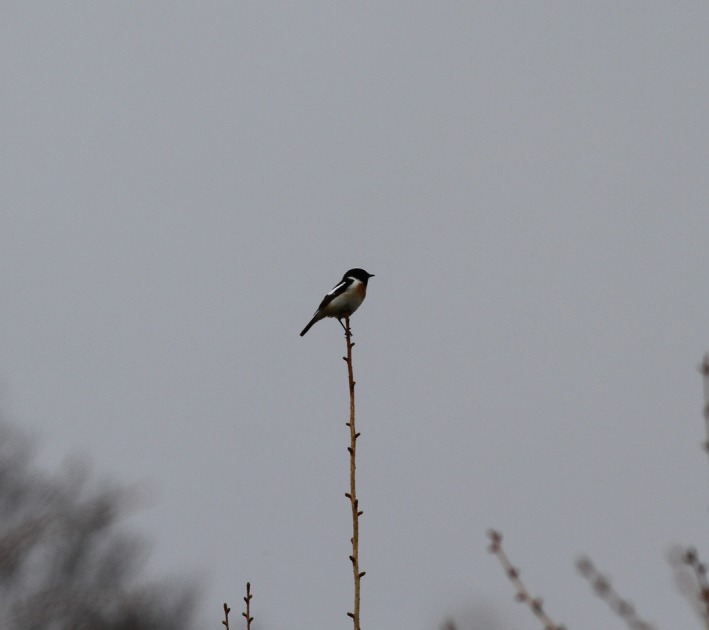
Stonechat *Saxicola torquata* – a representative early‐successional bird species – in a young larch *Larix leptolepis* plantation. A male individual perching on a planted larch.

We have long known that the detection probability of individuals is less than one and varies among species even for sessile plant species (Royle and Dorazio 2008; Chen et al. 2013). For species to be detected and enumerated in SARs requires that individuals of species present in the study area also be present in the subregions of the study area that are actually sampled (Fig. 1). Unless the study area is sampled completely, we must estimate the number of species present in the unsampled area in addition to the number of species undetected in the area sampled. Therefore, the estimation of species richness should play a prominent role in constructing SARs. The problem of estimating species richness has been addressed using a variety of methods (Gotelli and Colwell 2001; Mao and Colwell 2005; Hortal et al. 2006; Gotelli and Chao 2013), but approaches developed in that context have seldom been applied nor have they been adapted to the problem of estimating species richness in defined areal units such as habitat or true islands. One approach to SARs using these estimators is to estimate species richness in each area first and then regress these estimates against area (Borges et al. 2009). However, this strategy of doing “statistics on statistics” uses the estimates from the first step as “true” values without incorporating the uncertainty in the estimates in the second step (Royle and Dorazio 2008). Furthermore, these traditional estimators assume that net probability of detecting a species is due to the relative abundance of species and do not consider the role of heterogeneity of detection probability among species (Iknayan et al. 2014).

**Figure 2:**
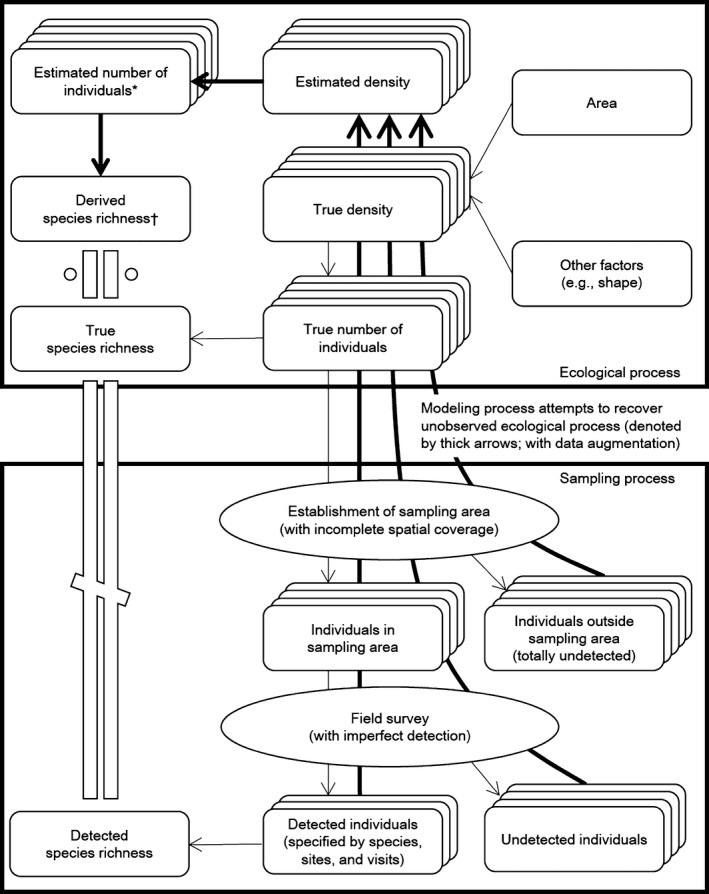
Conceptual framework of ecological and sampling processes involved in modeling species–area relationships (SARs). Abundance of individual species in the area of interest is determined by their densities and its area, and true species richness is a consequence of these abundances. Unless sampling plots cover the area entirely, only individuals in the sampling plots are exposed to sampling. During field surveys, some individuals may be undetected because of imperfect detection. SARs are traditionally estimated using only detected species. In this study, we propose a sampling model to consider these two sources of incomplete sampling separately. To account for unobserved species due to incomplete sampling, “potential” species with zero detected individuals are augmented, and combined detection histories of detected and potential species are analyzed to estimate abundances of individual species (including unobserved species) in each area (denoted by “*”). Our estimate of species richness is obtained as a derived parameter (†, i.e., the posterior distribution of the number of species with at least one individual). Based on these quantities across the species (including unobserved species), the total abundance of communities and the species richness are estimated.

Here, we propose a framework to model SARs accounting for incomplete sampling using hierarchical community models (Royle and Dorazio 2008; Iknayan et al. 2014). Hierarchical community models are ensembles of species‐level models from which community‐level state variables such as species richness can be derived (Royle and Dorazio 2008). Hierarchical community models contain both a model for the ecological process of interest, the abundances or frequencies of individual species at each site, and a model for the sampling process by which the data were generated. The central concept of our approach is to simultaneously estimate SARs and abundances or frequencies of individual species, using a model that accounts for the imperfect detection of individuals in the sampled area and the incomplete spatial coverage of the study area by sampling plots. Furthermore, we consider the contributions to SARs of species undetected throughout the survey using data augmentation (Royle and Dorazio 2008) (see Fig. 2 for a conceptualization of our modeling framework). Because hierarchical community models can include species‐level covariates, our modeling framework can relax the assumption of constant density. For example, we can allow for positive or negative density–area relationships (DARs) for individual species, which prevail in many landscapes (Bender et al. 1998; Connor et al. 2000; Brotons et al. 2003).

The goal of our study was first to outline an approach to model SARs subject to incomplete sampling using a class of multispecies abundance models. The second goal was to outline an approach to modeling SARs using a multispecies frequency model, specifically developed for plant surveys. We apply these models to data sets of early‐successional birds and plants in young larch plantations. While our data sets are not large, they allow us to model SARs accounting for incomplete sampling and thus serve as proof of concept.

## Materials and Methods

### An ecological process model for abundances

We propose a Poisson model for the latent patch‐level species abundance, *z*_*ij*_, in which the abundance of species *i* in patch *j* is as follows:

(1) $$z_{ij}∼\text{Poisson}\;(\lambda_{ij})$$

where *λ*_*ij*_ is expected abundance in the study patch. Because the model underlying eqn (1) does not explicitly account for the effect of area, we model the area dependence of abundance for each species (indirectly, area dependence of density) as the coefficient (*β*_1*i*_) of log‐transformed area (*A*_*j*_) using the log link (Connor et al. 1997):

(2) $$\log\;\left(\lambda_{ij}\right)=\beta_{0i}+\beta_{1i}\times \log\;\left(A_{j}\right)$$

where *β*_0*i*_ is logarithmically transformed abundance when area is 1 (if *A*_*j*_ = 1, then log(*A*_*j*_) = 0, and *λ*_*ij*_ = exp(*β*_0*i*_)). We make *β*_1*i*_ a free parameter rather than fixing it as 1 to directly test hypotheses of area dependence of the densities of individual species (Connor et al. 1997).

Our model assumes that variation in abundance can be fully explained by area. However, environmental differences among the patches could affect abundances so we can expand eqn (2) by including additional covariates:

(3) $$\log\;\left(\lambda_{ij}\right)=\beta_{0i}+\beta_{1i}\times \log\;\left(A_{j}\right)+x_{j}^{\prime }\beta_{i}+b_{ij}$$

where $x_{j}^{\prime }$ and ***β***_***i***_ are the patch‐specific covariate(s) and their coefficient(s), respectively. Random site effects (*b*_*ij*_) can be included to consider variation in abundance not captured by area, environmental covariates, and the associated Poisson distribution (Yamaura et al. 2012) and are assumed to be normally distributed:

(4) $$b_{ij}∼\text{Normal}(0,\sigma_{b}^{2}).$$

Although the single normal distribution of site effects is shared by all species, we could use species‐specific standard deviations when sufficient data are available (Yamaura et al. 2012). The negative binomial distribution rather than random site effects could be used to account for extra‐Poisson variation in abundance (Joseph et al. 2009). However, rather than using the negative binomial, we suggest that other approaches that examine the effects of unmodeled environmental covariates or intra‐ or interspecific associations among species might be more productive (Dorazio and Connor 2014; Dorazio et al. 2015).

It is difficult to analyze rare species with traditional statistical models because of their low detection frequencies. This is particularly important in SARs as rare species may dominate SARs (e.g., Patterson 1987). Using the idea of hierarchical community modeling, we assume that species‐level parameters (*β*_0*i*_ and *β*_1*i*_) have normal distributions shared by all species (including rare species):

(5) $$\beta_{0i}∼\text{Normal}\;(\mu_{\beta_{0}},\sigma_{\beta_{0}}^{2}),\;\beta_{1i}∼\text{Normal}\;(\mu_{\beta_{1}},\sigma_{\beta_{1}}^{2})$$

where $\mu_{\beta_{0}}$ is the mean value of *β*_0*i*_, and $\sigma_{\beta_{0}}$ is its standard deviation. In this way, we can model parameters of rare species, including species that are unobserved during the survey with data augmentation, by borrowing information from those of common species which are more reliably estimated (Royle and Dorazio 2008) (see section on Estimating species richness from abundance and frequency below).

### A sampling process model for abundances

We next relate observations obtained from a sampling protocol to the underlying true state variables, that is, abundances. In our case, we specify two specific observation models to account for the fact that sampling plots seldom completely cover the study area (incomplete spatial coverage) and the imperfect detection of individuals in the sampling plots. To address the issue of incomplete spatial coverage, we can relate the number of individuals exposed to sampling (*N*_*ij*_) to the true species abundance in patch *j* by assuming that *N*_*ij*_ increases proportionally to the ratio of sampling area to patch area. In other words, we assume that *N*_*ij*_ is a binomial random variable with probability parameter *ϕ*_*j*_:

(6) $$N_{ij}∼\text{Binomial}\;(z_{ij},\phi_{j})$$

where *ϕ*_*j*_ is obtained by dividing sampled area by patch area.

To consider imperfect detection, we can use different detection models depending on the survey methods. When we record simple counts of individuals, a binomial sampling model is reasonable (Yamaura et al. 2012; Dorazio and Connor 2014): *y*_*ijt*_ ~ Binomial(*N*_*ij*_,*p*_*i*_) where *y*_*ijt*_ is the number of individuals of species *i* in patch *j* detected on visit *t* and *p*_*i*_ is the probability of detecting an individual of species *i* conditional on presence. However, for our bird data, we have encounter histories of individuals over *t* visits (detection/nondetection of each individual on each visit). Therefore, we model the number of individuals detected with a multinomial mixture model in which the vector (of length *t *+* *1) of encounter frequencies ${\left\{y_{ij,H}\right\}}_{H=0}^{t}$ (where the vector is the number of individuals of species *i* in patch *j* that were detected *H *=* *0, 1, 2, 3,…, *t* times) is multinomial with cell probabilities ${\left\{\pi_{i,H}\right\}}_{H=0}^{t}$ (Royle et al. 2007bb):

(7) $${\left\{y_{ij,H}\right\}}_{H=0}^{t}∼\text{Multinomial}\;(N_{ij},{\left\{\pi_{i,H}\right\}}_{H=0}^{t}),$$

where the multinomial probabilities $\pi_{i,H}$ are functions of individual detection probability parameters, *p*_*i*_, that vary among species. By convention, the “0 cell” having probability *π*_0_ corresponds to the number of individuals not detected from among the community of species exposed to sampling. We use a re‐parameterization of the multinomial model in terms of the observed frequencies only, by “conditioning on encounter” (Appendix S1‐1).

### An ecological process model for frequencies

We propose another ecological process model, a frequency model, in which we use frequency (number of sampling plots in which an individual species occurs) rather than abundance because plant surveys usually record the occurrence of species (binary presence/absence) in regular‐sized sampling plots (e.g., Whittaker 1956). Species present in sampling plots are treated equally regardless of their abundances in the plots, and frequencies are usually much lower than abundances (Magurran 1988). We assume that patch‐level frequency (*z*_*ij*_) follows a binomial distribution with plot‐level occurrence probability (*ψ*_*ij*_) in each patch:

(8) $$z_{ij}∼\text{Binomial}\;(tp_{j},\psi_{ij})$$

where *tp*_*j*_ is the number of plots that tessellate the patch given that the whole patch is divided into equal‐sized plots. In that case, we model the area dependence of plot‐level occurrence probability in each patch using the logit‐link:

(9) $$\text{logit}\;\left(\psi_{ij}\right)=\beta_{0i}+\beta_{1i}\times A_{j}+x_{j}^{\prime }\beta_{i}+b_{ij}$$

where parameters are the same as in eqn (3) except that area is not logarithmically transformed. In this model, values of *β*_1*i*_ = 0 would suggest that the *per area* occurrence probability of an individual species is constant for all size areas, and tests could be applied to examine this assumption. We note that frequency and abundance are different quantities, and DARs and occurrence probability–area relationships may not be similar.

### A sampling process model for frequencies

In a typical plant survey, patches are only partially covered by sampling plots. To account for incomplete spatial coverage, we propose a sampling model linking the observed occurrence frequency (e.g., number of occurrences among the sampled plots) in each patch (*y*_*ij*_) to *ψ*_*ij*_ (Yamaura et al. 2012):

(10) $$y_{ij}∼\text{Binomial}\;(ap_{j},\psi_{ij})$$

where *ap*_*j*_ is the actual number of sampling plots in patch *j* and *ap*_*j*_ ≤ *tp*_*j*_. For our plant data, we assume perfect detection as is usually assumed in plant surveys. However, one could assume imperfect detection if plots are visited more than once and occupancy models are used (Royle and Dorazio 2008). We outline in Appendix S1‐2 the analogous model assuming imperfect detection.

We then estimate the occurrence frequency of each species in the nonsurveyed portion of each patch *z*(*tp* – *ap*)_*ij*_: *z*(*tp* – *ap*)_*ij*_ ~ Binomial([*tp* – *ap*]_*j*_,*ψ*_*ij*_) where (*tp* – *ap*)_*j*_ is the number of nonsurveyed plots. If, for example, we use 1‐m^2^ plots and measure patch area in ha, (*tp* – *ap*)_*j*_ = 10,000 × *A*_*j*_ – *ap*_*j*_. We generate patch‐level occurrence frequency (*z*_*ij*_) by adding *y*_*ij*_ to *z(tp* – *ap)*_*ij*_. Using this model structure, we assume that there are species that only occur in the nonsampled area (i.e., species with *z*_*ij*_ > 0 but *y*_*ij*_ = 0). We can estimate the number of species not encountered in each patch and across patches due to incomplete sampling using the data augmentation technique described below (i.e., we augment potential species with zero observed frequency in every patch).

### Estimating species richness from abundance and frequency

A key aspect of formal inference about biological communities from field survey data is that we cannot expect to observe all of the species in the community, *S*. While our ecological process models described above apply to all species in the community, our sampling of the community yields data that is biased to favor species that are both more abundant or more frequent and also more highly detectable. Species that go undetected by the field survey, either because they occur in the region outside the sampled patches, or because they occur only in areas of the patches that were not covered by sampling plots, or because they occur on sampling plots but were undetected, exist as all‐zero encounter histories, and it is unknown how many of such all‐zero encounter histories there are at each sampled location, and indeed among all sampled locations. To account for this realistic situation, we put a prior distribution on the unknown quantity *S* and treat it as a parameter to be estimated along with the other structural parameters of the model (the coefficients of the covariates and so forth).

The fact that *S* is an unknown parameter that must be estimated induces some special difficulties in the fitting of such models. Namely, the number of parameters of the model itself is an unknown quantity. That is, because we assume that abundances or frequencies and detection probabilities are different for each species in the community, the number of such species‐specific parameters is a multiple of *S*. This problem of a “variable dimension parameter space” has received much attention in the statistical literature where it is commonly addressed by the method of Reversible Jump Markov chain Monte Carlo sampling MCMC (King and Brooks 2008; Gimenez et al. 2009; King et al. 2010), and also the method of data augmentation (Royle et al. 2007a). We adopt the method of data augmentation here as in the models we have previously developed (Yamaura et al. 2011, 2012, 2016).

A heuristic explanation of data augmentation is as follows: we know that unobserved species in our sample must possess an “all‐zero” encounter history. Therefore, we add to our data set a large but fixed number, *M*, of artificial species with all‐zero encounter histories. We estimate the proportion (Ω) of these species, which are exposed/available to the field survey, among the augmented *M* species – the “presence” species. That is, instead of estimating *S*, the unknown size of the true community, we fix its upper limit at *M* and estimate the parameter Ω. The key technical aspect of data augmentation is that estimation of Ω (for fixed *M*) and estimation of *S* are statistically equivalent problems (Royle et al. 2007a; Royle and Dorazio 2012), but the former is somewhat easier to deal with in practice, especially using modern computing software for Bayesian analysis (BUGS, JAGS). Specifically, we can formulate whether an individual species is a member of the community using an indicator variable, *w*_*i*_ *,* and the Bernoulli distribution: *w*_*i*_ ~ Bernoulli(Ω). We then modify the ecological Poisson process model of abundance: $z_{ij}∼\text{Poisson}(wi\times \lambda_{ij})$ or the binomial process model of frequency: $z_{ij}∼\text{Binomial}(w_{i}\times \psi_{ij})$. This formulation leads to structural zeros for encounter histories (*y*_*ij*_ = 0) of the species that are not incorporated in the community (as *w*_*i*_ = 0 and therefore *z*_*ij*_ is always zero). However, for species incorporated in the community (*z*_*ij*_ ≥ 0 and *w*_*i*_ = 1), abundance and detection histories of unobserved species are *sampling* zeros, rather than *structural* zeros.

In our model, *S* is the number of species in the community that was sampled. Our explicit sampling model includes two levels of sampling: a sample of patches is selected from the region harboring the community of *S* species. Secondly, in each patch, we carry out a survey to detect and count species. Therefore, the number of unobserved species (species with detection histories of all *y*_*ij*_ = 0), which we aim to estimate, includes two classes of species; first, there are species that occur on the sampled patches but that went undetected by the survey activity in the sampling plots within those patches; second, there are species that occur in the community of *S* species but do not occur on any of the sampled patches. Note that we do not include the number of the first class of unobserved species as an explicit parameter in the model but obtain it as a derived parameter because it is completely determined by the parameter *S* and also the individual species‐level abundance states. That is, by estimating abundance or frequency, *z*_*ij*_, for species *i* in the community at sampled patch *j* and then summing up the number of species that exist (have *z*_*ij*_ > 0), we can tabulate the number of species occurring on the sampled patches. See Appendix S1‐3 for alternative approaches to estimating patch‐specific species richness.

We can obtain the number of the second class of unobserved species as the number of species that do not occur on the sampling plots (have *z*_*ij*_ = 0 for all patches) but are incorporated in the community (have *w*_*i*_ = 1). Therefore, we can distinguish between these two types of undetected species because our sampling model deals with them explicitly. On the other hand, sometimes the sampled patches do not represent an explicit sample from some well‐defined landscape and therefore, in general, the parameter *S* is mainly an abstract quantity representing the number of species that exist in some large landscape of which our sample is representative (Kéry and Royle 2009). Under the model, for a fixed value of *S*, if we made predictions for a set of patches, then the predicted number of species on that set of patches would increase to *S* as we increased the number or area of such patches.

### Study area and field sampling

We conducted field surveys of young larch plantation patches in the eastern Tokachi plain (Urahoro and Ikeda town), eastern Hokkaido, northern Japan (42°54′N, 143°36′E). We selected 13 young larch plantation patches varying in size from 1.3 to 10 ha (Appendix S2). All patches were surrounded by mature natural or plantation forests, were more than 35 m from other open land uses (e.g., arable fields), and were separated by at least 1.6 km from each other. All patches were 4–6 years old and created by cutting mature plantations and re‐planting larches.

We surveyed birds using territory mapping during the breeding season of 2011 (Bibby et al. 2000). A single observer (Y.Y.) visited each patch five times walking a 100‐m‐wide transect that covered the entire area of each patch. We clustered detections into putative territories based on territorial conflicts, other behavioral observations, and knowledge of territory size to create encounter histories describing the pattern of detection (*y *=* *1) or nondetection (*y *=* *0) for each territory. For example, the encounter history [00100] indicates a territory that was only detected during the third visit. We counted the number of territories detected one to five times at each patch for each species, ${\left\{y_{ij,H}\right\}}_{H=1}^{5}$.

We surveyed plants during the summer of 2011 by establishing a square grid of 1 × 1 m^2^ plots spaced 25 m apart for each patch. The number of sampling plots per patch varied from 20 to 161 depending on patch area. We recorded the plant species occurring in each plot, excluding planted larches. Most patches had a sample density of approximately 16 plots per ha. However, in three patches that were partially weeded prior to the survey, we only established plots in the unweeded area (75–90% of patch area: Appendix S2).

We categorized bird species into early‐successional and mature forest species and plant species into early‐successional, mature forest, and exotic species based on previous studies and local expert opinion (Yamaura et al. 2012). Species in the same group may have similar responses to patch area. We treated exotic species as a single group. For birds, we only used data on early‐successional species as they nested and foraged within the patches. Only a few transient individuals of mature forest species entered the patches.

### Model applications to the data

We fit abundance and frequency models to bird and plant data, respectively. We used patch area as the only covariate for both taxa. However, we included random site effects because factors other than patch area (e.g., stand age and topography) could still affect the distributions of individual species. For birds, we used the number of territories as an index of bird abundance and did not consider incomplete spatial coverage because our survey transects covered all patches entirely (*N*_*ij*_ = *z*_*ij*_). For plants, we assumed perfect detection because we tried to identify all species in the plots irrespective of time (e.g., we took more than 20 min per species‐rich plot: Yamaura et al. 2012). Because we categorized plant species into one of three groups, we used separate normal distributions with group‐specific hyperparameters for intercepts and slopes (Yamaura et al. 2012), for example, $\beta_{1i}∼\text{Normal}\;(\mu_{\beta_{1,\mathrm{group}[i]}},\sigma_{\beta_{1,\mathrm{group}[i]}}^{2})$. We assigned undetected species estimated to be present into one of the groups based on the observed proportions of species detected in each group using a Dirichlet distribution (Yamaura et al. 2011, 2012: see Appendix S1‐4 for details). We then model their occurrence probabilities and frequencies with group‐level hyperparameters.

### Parameter estimation and model assessment

We estimated model parameters by computing posterior distributions using Markov chain Monte Carlo sampling (MCMC) with JAGS ver. 3.2.0 (Plummer 2012), R2jags ver. 0.03‐08 (Su and Yajima 2012), and R ver. 2.14.1 (R Development Core Team 2012) (see Appendix S1‐5 for details). We augmented the plant data set with 1000 potential species that might have been present yet undetected. These numbers have to be larger than potential numbers of undetected species, but not so large as to unnecessarily extend computation time (Royle and Dorazio 2008). Because the posterior distribution of inclusion probability (Ω), which indicates the proportion of species that would be present among the augmented species, was well below 1 (median <0.52), the number of augmented plant species was sufficiently large (Royle and Dorazio 2008). We obtained a similar estimate of species richness when we used 2000 potential species. However, for birds, the upper limit of the 95% CI reached 1 and suggested that the regional species pool included an additional 32 early‐successional bird species (50 potential species were added). Nevertheless, based on expert knowledge, few if any early‐successional bird species that could occur in early‐successional forests in this region were undetected (Y. Yamaura, pers. obs.). Therefore, we fit the abundance model to the bird data without using data augmentation.

We estimated patch‐specific bird abundance and plant frequency of all species for patches with areas ranging from 0.01 to 10 ha for birds and 0.0001 to 10 ha for plants using the multispecies models described above with random site effects. Given the predicted abundance or frequency of individual species, we enumerated the number of species predicted to occur (i.e., have at least one individual) in each patch, that is, we obtained the estimates of species richness for each patch as a derived parameter. These estimates account for individuals present but undetected in the sampling plots and for individuals in the areas of the patches that were not subject to sampling for both detected and undetected species. We also obtained estimates of group‐specific frequency and species richness for plants.

It is not straightforward to test the effects of DARs on the form of SARs and abundance–area relationships even using the community‐level hyperparameters because community‐level properties (e.g., species richness, total abundance) are derived parameters. We assessed the effects of DARs on SARs and abundance/frequency–area relationships by fitting the abundance model with *β*_1*i*_ = 1 and the frequency model *β*_1*i*_ = 0 for all species and with standard deviations = 0. We call these models “null models,” and this procedure is equivalent to comparing the constant density or occurrence probability hypothesis to a hypothesis in which *β*_1*i*_ is a free parameter (see Appendix S1‐6 for details). To make these “null models” strictly comparable to the models we fit to the data, we included random site effects as these were also included in the fitted models. We also conducted a set of simulations to confirm our suspicion that the aggregate patterns of DARs within a community could affect the form of SARs (see Appendix S3 for details).

## Results

### Abundance model for birds

**Figure 3:**
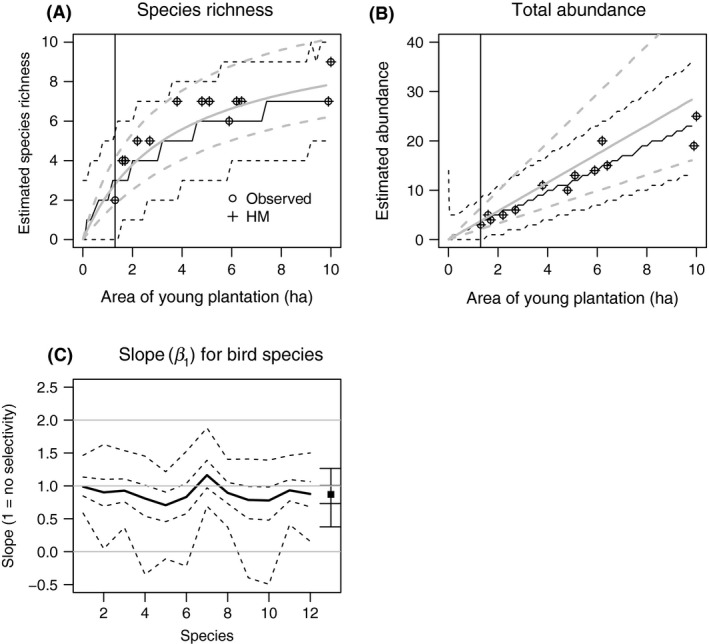
Species richness, total abundance, and *β*_1*i*_ values for early‐successional bird species in larch plantation patches. (A) Species richness and (B) total community abundance as a function of patch area. Solid and dotted black lines indicate the median and 95% CIs derived from multispecies abundance model (HM), respectively. Vertical line indicates the smallest area of our sampled patches. Estimated values smaller than this area are derived from extrapolation of the model. Solid and dotted gray lines were predictions from null models under constant density hypothesis. Due to high detection probability of bird species, observed species richness and abundances were equal to their estimated values. (C) Estimated values of *β*_1*i*_ from the abundance model for each species. Solid line is the median and the inner and outer dotted lines are the 50% and 95% CIs, respectively. The rightmost box and vertical bar indicate the median, 50%, and 95% CIs of the estimated community‐level hyperparameter (mean value of *β*_1*i*_ across species).

Although we observed 39 bird species within patches and in mature forest adjacent to patches, mature forest species were transient and rarely detected on more than one visit. We encountered 150 territories of the 12 strictly early‐successional species. Community‐level detectability (individual‐level detection probability averaged across species, $\overset{¯}{p}$), which was derived from the posterior median of the hyperparameter, was 0.66 (0.53–0.73). This suggests that each territory would be detected at least once if sites were visited five times (>99%). Indeed, estimated species richness and community‐level (total) abundance at each site were not different from the observed values (Fig. 3A,B). Estimated species richness increased with patch area as a saturating curve. The confidence intervals for the effect of area at individual and community levels (*β*_1*i*_ and ${\overset{¯}{\beta }}_{1}$) included 1, indicating that all of these bird species showed no dependence of density on patch area (Fig. 3C). Indeed, predicted values of species richness and total abundance from multispecies models were quite similar to those from null models assuming constant density (Fig. 3A,B).

### Frequency model for plants

**Figure 4:**
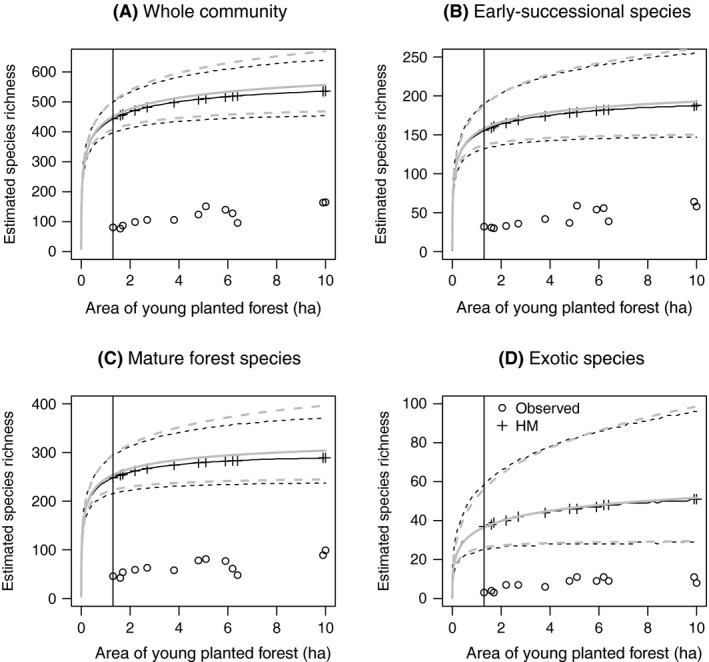
Plant species richness in relation to area of larch patches for (A) the entire plant community, (B) early‐successional species, (C) mature forest species, and (D) exotic species. Estimated values were derived from the multispecies frequency model. See Figure 3 for detailed descriptions of symbols. Four figures have different ranges of vertical axes. Although we encountered 314 species throughout the survey, we only observed a subset of these species in each patch because of the field survey did not cover the entire area of each patch (incomplete spatial coverage). Hence, our estimated values for species richness which account for incomplete spatial coverage are substantially higher than the observed values of species richness.

We identified 314 plant species in the field and grouped them into 114 early‐successional, 177 mature forest, and 23 exotic species. The regional species richness (*S*) was estimated to be 689 (503–1095), indicating that we would encounter an additional 375 (189–781) species if we surveyed most of this habitat in the region. When species richness is estimated accounting for incomplete spatial coverage, richness estimates are substantially higher than naive estimates simply based on the number of species detected in each patch (Fig. 4). This is because the naive estimates are strongly biased underestimates of species richness. In contrast to bird species richness, plant species richness was predicted to depend very weakly on patch area across the sampled range of patch areas (Fig. 4). Model extrapolation to patches smaller than the sampled patches suggested that species richness would decline as expected in very small patches (<1 ha).

**Figure 5:**
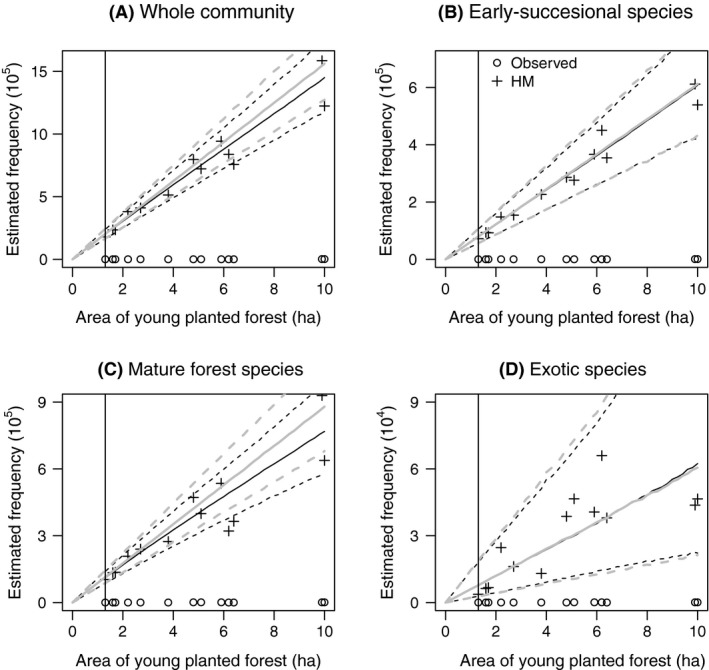
Plant frequency in relation to area of larch patches for (A) the whole plant community, (B) early‐successional species, (C) mature forest species, and (D) exotic species. Estimated values were derived from the frequency model. See Figure 3 for detailed description of symbols. Four figures have different ranges of vertical axes. Note again that estimated frequencies under our model are substantially higher than the observed frequencies as the model‐derived estimates account for incomplete spatial coverage.

**Figure 6:**
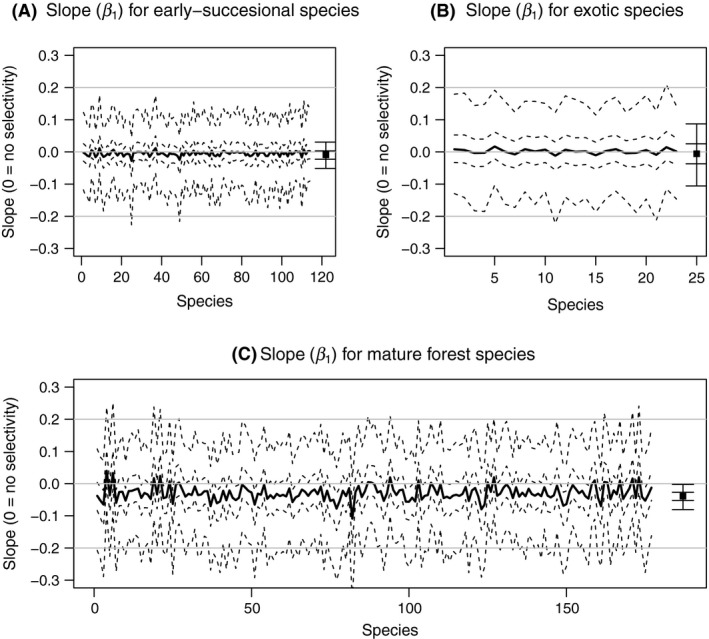
Estimated values of *β*_1*i*_ from the plant frequency model for (A) early‐successional species, (B) exotic species, (C) and mature forest species. See Figure 3 for detailed descriptions of the symbols.

Neither community‐ or group‐level SARs or frequency–area relationships deviated from predictions of null models (Figs 4 and 5), and neither early‐successional or exotic species showed area dependence of occurrence probability (Fig. 6). However, for many mature forest plant species, the posterior distribution of *β*_1*i*_ slightly favored negative values, and the group‐level mean value (${\overset{¯}{\beta }}_{1}$) was significantly less than 0 (Fig. 6C). This indicates that many mature forest plant species collectively showed a slight tendency to be more common in small patches. Nevertheless, predicted SARs and frequency–area relationships of mature forest plant species were almost the same as those from null models (Figs 4C and 5C), indicating that these negative occurrence probability–area relationships were not strong enough to change the form of SARs and frequency–area relationships.

## Discussion

### Modeling SARs subject to incomplete sampling

Species–area relationships are consequences of ecological processes by which species with at least one individual are added into communities as patch area increases. We model SARs by modeling the patch‐specific abundance or frequency of each species. Echoing the call to separate sampling processes from ecological processes (Cam et al. 2002), we estimated SARs with an explicit sampling model to account for imperfect detection and incomplete spatial coverage separately. Our models account for the sampling process by accommodating the imperfect detection of individuals within the sampling plots, and the individuals ignored in areas of the patch not covered by sampling plots. Previous attempts to model SARs that have not explicitly accounted for these two forms of sampling incompleteness almost certainly underestimate species richness. Our approach requires an explicit sampling model and a sampling design that includes repeated observations on each study patch to account for imperfect detection. It is not an alternative way to analyze legacy SAR data as most of these data sets were generated without using a specific sampling design, without estimating patch and species‐specific abundances, and without making repeated observations on each study patch.

The modeling approach we propose can accommodate a variety of sampling methods by adopting appropriate models of the sampling process. In this study, we adopted a territory mapping method for birds and considered imperfect detection using a capture–recapture model. However, one of the most widely adopted sampling methods is counting “unmarked” individuals from repeated visits, and we can treat this type of data with a binomial mixture model (Yamaura et al. 2012; Dorazio and Connor 2014). As we also develop a multispecies frequency model, we can deal with binary plot data in which we record species occurring in each plot. Other sampling methods can be accommodated by application of an appropriate sampling model.

We modeled community assembly as a summation of an ensemble of species‐level Poisson or binomial processes and estimate a scaling parameter linking abundance or frequency of individual species and patch area from the data, rather than treating it as a fixed parameter. Community‐level properties including species richness and its dependence on area (SARs and abundance/frequency–area relationships) are obtained as derived parameters of a hierarchical model. Models of SARs under the random placement hypothesis (Arrhenius 1921; Coleman et al. 1982) are simply special cases of the Poisson abundance model that we propose with the scaling parameter *β*_1*i*_ equal to 1. However, unlike the random placement model, we treat the *z*_*ij*_ (species‐ and patch‐specific abundances or frequencies) as estimable parameters subject to sampling, rather than as fixed known values. Given that our model is extensible and other covariates could be included, it will be productive to examine their effects on community‐level properties.

Accounting for the existence of undetected species throughout the study area is also at the core of our approach to modeling SARs. We used parameter expanded data augmentation to allow us to estimate the number of undetected species in each patch and in the set of patches, as do traditional species richness estimators (see Appendix S4 for a comparison of species richness estimates from our multispecies models and traditional species richness estimators). Model application to the plant data suggests that 375 (95% CI: 189–781) plant species would be undetected among the regional species pool, which is reasonable because most plant species were rarely encountered. Indeed, based on the knowledge of the regional flora, we expect that approximately 400 additional plant species could occur in these habitats (K. Ito, pers. obs.). We note that our plant survey only covered 0.14% of the total area of the study patches, and there were large differences between observed and estimated values for plant species richness and plant species frequencies (Figs 4 and 5). On the other hand, data augmentation did not perform well in estimating the species richness for birds. A possible explanation for this failure is that the number of undetected species was nonidentifiable due either to the sparse data or excessive heterogeneity among the species (Dorazio and Royle 2003). For example, we only detected 12 species, and many of them were rarely detected. One solution in this case may be to fix the upper bound of species richness from expert knowledge of the regional species pool (Dorazio et al. 2011).

### Relaxation of constant density hypothesis

Although existing models of SARs assume that densities of individual species are constant (Preston 1962; MacArthur and Wilson 1967; May 1975; Coleman et al. 1982; Williams 1995), the literature has increasingly shown that individual species display a variety of DARs (Bender et al. 1998; Connor et al. 2000; Brotons et al. 2003). We relaxed the constant density hypothesis in our models and also conducted simulation experiments (Appendix S3). While our plant and bird data did not show that DARs affect the form of SARs, this is expected given the small size and narrow range of patch sizes in our field study. However, results from the simulations clearly showed that the aggregate pattern of individual species DARs and occurrence probability–area relationships can alter the form of SARs. In general, communities with greater proportions of species with negative DARs (edge species) or occurrence probability–area relationships tend to have SARs with lower slopes (Appendix S3).

Model application to the field data found no bird species preferred large patches (*β*_1*i*_ = 1) (Fig. 3C), suggesting that for these species small patches have the same value per unit area as large patches. However, for birds, our data focused on early‐successional species in small plantation patches (<10 ha), so it is not surprising that bird species showed no area dependence of densities unlike studies on birds that covered a wider range of patch areas (Guadagnin et al. 2009; Bidwell et al. 2014; Dorazio and Connor 2014). On the other hand, we found that mature forest plant species as a group showed slightly higher occurrence probabilities in small patches (Fig. 6), which may be due to the existence of positive edge effects and immigration from surrounding mature forests (Bowman et al. 2002). However, comparisons between the SARs estimated from our models and those generated under null models assuming constant occurrence probability suggest that the observed deviations were not large enough to alter the form of SARs (Figs 4 and 5).

## Conclusions

By modeling community assembly as the summation of an ensemble of species‐level Poisson or binomial processes, we have attempted to explicitly unify the study of species‐ and community‐level patterns of abundance/frequency and species richness. Our approach perceives the estimation of SARs as a problem of accurately and efficiently estimating the abundances or frequencies of each species in each study patch. The desire to unify species‐ and community‐level processes has long been a theme in ecology (Preston 1962; MacArthur and Wilson 1967; May 1975; Coleman et al. 1982; Williams 1995; He and Legendre 2002; Ovaskainen and Hanski 2003). As it has long been known that complete sampling is rarely attained, we suggest that without considering the sampling processes that generate the data such unification would remain problematic.

While the approach we propose requires substantially more data to implement than has historically been used to estimate SARs, it yields a much richer array of information about the scaling of species abundances or frequencies, and species richness with area. Most importantly, our approach explicitly separates sampling processes from the estimation of ecological processes and by doing so should yield a clearer picture of the ecology that underlies SARs and DARs.

## Data availability

Details of sampling sites, sampled species, and results of the analysis are available from Pangaea data base: http://doi.pangaea.de/10.1594/PANGAEA.841125.

## Conflict of Interest

None declared.

## Supporting information


**Appendix S1.** Full description of model development accounting for incomplete sampling.

**Appendix S2.** Study area and details of sampling sites.

**Appendix S3.** Simulation experiments on the roles of density‐area relationships on species‐area relationships

**Appendix S4.** Comparison between multispecies models and traditional estimators using field data and simulation experiments.

**Appendix S5.** A zip file composed of R scripts and the source data to be analyzed.
