# Supplementary material for: Estimating species – area relationships by modeling abundance and frequency subject to incomplete sampling
Source: Ecol Evol. 2016 Jun 17;6(14):4836–48. doi: 10.1002/ece3.2244 (PMC4979711; doi:10.1002/ece3.2244)
Supplement: Supplementary file 1 — Appendix S1. Full description of model development accounting for incomplete sampling. [file ECE3-6-4836-s001.docx]

**Appendix S1. Full description of model development accounting for incomplete sampling.**

1. A formulation of multinomial detection model

To consider imperfect detection, we can use different detection models depending on the survey methods. When we record simple counts of detected individuals, we can use a binomial observation model ([Yamaura *et al.* 2012](#_ENREF_14); [Barnagaud *et al.* 2014](#_ENREF_2)): *y_ijt_* ~ Binomial(*N_ij_*,*p_i_*) where *y_ijt_* is the number of detected individuals of species *i* in patch *j* and visit *t*. If we have encounter histories of individuals, such as from a capture-recapture, multiple observer, or removal sampling protocol, we model the number of individuals detected with a multinomial model. For example, if we sample for five visits and obtain detection/non-detection observations of individuals (or territories) during each visit, then the resulting encounter histories have a multinomial distribution conditional on local population size. Here we assume the vector of encounter frequencies $\left\{ y_{ij,H} \right\}_{H=1}^{5}$ (number of individuals detected one to five times) is multinomial with cell probabilities $\left\{ \pi_{i,H} \right\}_{H=1}^{5}$ (Table S1-1: [Royle *et al.* 2007](#_ENREF_11); [Chandler *et al.* 2013](#_ENREF_3)):

$\left\{ y_{ij,H} \right\}_{H=1}^{5}\sim Multinomial\left( N_{ij},\left\{ \pi_{i,H} \right\}_{H=1}^{5} \right)$. eqn S1

The frequency of undetected individuals say is defined as the difference between and the total number of encountered individuals. Similarly, the multinomial cell probability representing the probability of not being encountered is 1 minus the sum of the remaining cell probabilities.

The vector of counts $\left\{ y_{ij,H} \right\}_{H=1}^{5}$ is the sufficient statistic for a capture-recapture model to estimate the number of individuals in a single species situation where encounter probability is heterogeneous among individuals – the so-called “model Mh” ([Otis *et al.* 1978](#_ENREF_9); [Royle *et al.* 2007](#_ENREF_11)). We use this modeling framework, and allow the encounter (or detection) probability to vary among species. However, to our knowledge, current Bayesian estimation programs do not allow the sample size of the multinomial distribution (here *N_ij_*) to be unknown. Therefore, we re-parameterized the multinomial distribution in which we model the number of individualsdetected *n_ij_* using a binomial distribution with the probability that each individual is detected at least once, *pdet_i_* = $\pi_{i,1}$ + $\pi_{i,2}$ + $\pi_{i,3}$ + $\pi_{i,4}$ + $\pi_{i,5}$:

*n_ij_* ~ Binomial(*N_ij_*, *pdet_i_*). eqn S2

We model $\left\{ y_{ij,H} \right\}_{H=1}^{5}$ in terms of the conditional multinomial observation model given *n_ij_*:

$\left\{ y_{ij,H}| n_{ij} \right\}_{H=1}^{5}\sim Multinomial\left( n_{ij},\left\{ \pi_{i,H}^{d} \right\}_{H=1}^{5} \right)$ eqn S3

where the conditional cell probabilities are $\left\{ \pi_{i,H}^{d} \right\}_{H=1}^{5}$ = $\left\{ \pi_{i,H} \right\}_{H=1}^{5}$ / *pdet_i_*.

Table 1. Multinomial cell probabilities for the bird abundance model. *p_i_* is an individual-level detection probability of species *i* ([Royle *et al.* 2007](#_ENREF_11); [Royle & Dorazio 2008](#_ENREF_10)).

| Number of detected times (*H*) | Cell probability ($\pi_{i,H}$) |
| --- | --- |
| 1 | 5 × $p_{i}^{1}$ (1 – *p_i_*)^4^ |
| 2 | 10 × $p_{i}^{2}$ (1 – *p_i_*)^3^ |
| 3 | 10 × $p_{i}^{3}$ (1 – *p_i_*)^2^ |
| 4 | 5 × $p_{i}^{4}$ (1 – *p_i_*)^1^ |
| 5 | 1 × $p_{i}^{5}$ (1 – *p_i_*)^0^ |

1. A frequency model with imperfect detection

In a typical plant survey patches are only partially covered by sampling plots. To account for incomplete spatial coverage, we propose a sampling model linking the occurrence state (presence/absence) at each sampling plot in each patch to $\psi_{ij}$ ([Yamaura *et al.* 2012](#_ENREF_14)):

$z_{ijk}\sim Bernoulli(\psi_{ij})$ eqn S4

where *z_ijk_* is a binary state variable indicating whether species *i* is present or not in sampling plot *k* in patch *j*. Here we simply assume that occurrence probability is constant across the plots in the same patch. Although perfect detection is usually assumed in plant surveys, one could assume imperfect detection if plots are visited more than once and occupancy models are used ([Royle & Dorazio 2008](#_ENREF_10); [Chen *et al.* 2013](#_ENREF_4)):

$y_{ijkv}\sim\mathrm{Bernoulli}(z_{ijk}\times p_{i})$ eqn S5

where *y_ijkv_* is also a binary detection/non-detection of species *i* in plot *k* in patch *j* on visit *v*, and here *p_i_* is plot-level detection probability of species *i*. We then estimate the occurrence frequency of the non-sampled plots of each patch (*z*(*tp* – *ap*)*_ij_*): *z*(*tp* – *ap*)*_ij_* ~ Binomial((*tp* – *ap*)*_j_*,* *$\psi_{ij}$) where (*tp* – *ap*)*_j_* is the number of non-surveyed plots. If, for example, we use 1 m^2^ plots, and measure patch area in ha, (*tp* – *ap*)*_j_* = 10,000 × *A_j_* – *ap_j_* where *ap_j_* is the number of sampling plots in patch *j*. We generate patch-level occurrence frequency (*z_ij_*) by adding $\sum_{k=1}^{{ap}_{j}} z_{ijk}$ to *z*(*tp* – *ap*)*_ij_*. Using this model structure, we assume that there are species that only occur in non-sampled areas (i.e., species with $\sum_{k=1}^{{ap}_{j}} z_{ijk}$ >0 but $\sum_{k=1}^{{(tp-ap)}_{j}} z_{ijk}$ > 0) and also species undetected but present in the surveyed plots (i.e., $\sum_{k=1}^{{ap}_{j}} \sum_{v=1}^{V_{j}} y_{ijkv}$ = 0 but $\sum_{k=1}^{{ap}_{j}} z_{ijk}$ >0). We can estimate the number of species not encountered at each patch and across patches due to incomplete sampling with the data augmentation technique (i.e., we augment potential species with zero observed frequency in every patch).

1. Expected values of species richness derived from abundance and frequency models

Althoguh we estimated patch-level species richness using the posterior distributions of number of species with at least one individual or frequency at each patch, we have alternative formulations of the expected values. These alternative formulations yield the same estimates of species richness as those from the posterior distributions of the species counts ([Yamaura, Kéry & Royle 2016](#_ENREF_12)); however, they may be useful to predict the species richness under the varied values of area and covariates.

- 1. Expected species richness of abundance model

In a random placememnt model proposed by [Arrhenius (1921)](#_ENREF_1) and [Coleman *et al.* (1982)](#_ENREF_5), the expected (or mean) species richness in patch *j* is

$E\left[ \text{species richness}_{j} \right]{=S-\sum}_{i=1}^{s}\left( 1-\alpha_{j} \right)^{z_{i}}$ eqn S6

where *α_j_* = *A_j_*/*A_t_*, *A_j_* is the area of the *j*th patch, $A_{t}=\sum_{j=1}^{J}A_{j}$is the sum of the areas of the *j* patches under study, *z_i_* is the overall abundance of species *i* in *A_t_*, and *S* is the number of species residing in *A_t_*. This model is equivalent to placing the *z_i_* individuals of species *i* into *j* patches independently and at random with probability *A_j_*/*A_t_* so that population densities are assumed to be independent of area, and overall abundances of individual species, *z_i_*, are treated as known quantities.

In this study, we propose a Poisson version of the random placement model (i.e., the abundance of species *i* in patch *j* is *z_ij_* ~ Poisson[*λ_ij_*]). Under the Poisson abundance model, the probability that at least one individual of a species occurs in an area is ([Royle & Dorazio 2008](#_ENREF_10)):

$Pr\left[ z\geq1 \right]=1-exp\left( -\lambda\right)$ eqn S7

where *λ* is expected abundance in the area.

If we expand this probability to multiple species and patches, expected species richness of patch *j* is obtained by summing up these probabilities across species

$E[{species richness}_{j}]=\sum_{i=1}^{S} \left[ 1-exp\left( -\lambda_{ij} \right) \right]$ eqn S8

where *λ_ij_* is the expected abundance of species *i* in patch *j*.

We model *λ_ij_* as a function of the area and the covariates using the log-link:

$\log\left( \lambda_{ij} \right)=\beta_{0i}+\beta_{1i}\times\log\left( A_{j} \right)+\boldsymbol{x}_{j}^{'}\boldsymbol{\beta}_{i}$ eqn S9

where ***x'_j_*** and $\boldsymbol{\beta}_{i}$ are the patch-specific covariate(s) and their coefficient(s), respectively.

We therefore formulate the expected values of species richenss as the amsemble of species-level models:

$E[{species richness}_{j}]=\sum_{i=1}^{S} \left[ 1-exp\left( -\exp\left( \beta_{0i}+\beta_{1i}\times\log\left( A_{j} \right)+\boldsymbol{x}_{j}^{'}\boldsymbol{\beta}_{i} \right) \right) \right]$. eqn S10

- 1. Expected species richness of frequency model

In our proposed frequency model, we formulate plot-level occurrence probability of individual species (*_ij_*) as follows:

$\mathrm{logit}\left( \psi_{ij} \right)=\beta_{0i}+\beta_{1i}\times A_{j}+\boldsymbol{x}_{j}^{'}\boldsymbol{\beta}_{i}$. eqn S11

We assume that patch-level frequency (*z_ij_*) follows a binomial distribution with plot-level occurrence probability in each patch: *z_ij_* ~ Binomial(*tp_j_*,*_ij_*) where *tp_j_* is the number of plots that tessellate the patch given that the whole patch is divided into equal-sized plots. On the basis of this species-level formulation, the probability that a species occurs in at least one plot in patch *j* can be obtained as the complement of the probability that the species does not occur in any plots ([Royle & Dorazio 2008](#_ENREF_10)):

$Pr\left[ z\geq1 \right]=1-\left( 1-\psi\right)^{{tp}_{j}}$. eqn S12

We model expected species richness as the function of patch area (via *tp_j_*) by summing up this probability across species:

$E\left[ {species richness}_{j} \right]=\sum_{i=1}^{S} \left[ 1-\left( 1-\psi_{ij} \right)^{{tp}_{j}} \right]$ eqn S13

where realized species richness is the number of species with frequency of at least one, given that their frequencies (*z_ij_*) follow a binomial distribution with parameters *tp_j_* and *_ij_*.

We note that we can directly link the occurrence probability to the expected abundance as in eqn S7. In this case, we model the area dependence of density as in eqn S9 by converting eqn S7 (here *Pr*[*z_ij_* ≥ 1] is denoted by *_ij_*) using a complementary log-log link function ([McCullagh & Nelder 1989](#_ENREF_8)):

$\log\left( -\log\left( 1-\psi_{ij} \right) \right)=\log\left( \lambda_{ij} \right)=\beta_{0i}+\beta_{1i}\times\log\left( A_{j} \right)+\boldsymbol{x}_{j}^{'}\boldsymbol{\beta}_{i}+b_{ij}$.

eqn S14

1. Use of Dirichlet distribution to assign undetected plant species into species groups

We estimated the number of undetected plant species due to incomplete sampling using data augmentation technique. We assigned these undetected species into one of the groups based on the observed proportions of number of detected species among groups with a Dirichlet distribution ([Yamaura *et al.* 2011](#_ENREF_13); [Yamaura *et al.* 2012](#_ENREF_14)). We used this distribution because the summation of proportions of individual groups was 1. For example, an undetected plant species was assigned into one of the three groups, and assignment probabilities of these three groups summed to 1. To do this, we introduced a species-level parameter, *G_i_*, the group membership of species *i*, which is unobserved for undetected species. We determined the value of *G_i_* for detected species based on previous studies and local expert opinion. Specifically, for plants, *G_i_* = 1, 2, 3 for early-successional, mature forest, and exotic species, respectively. We assumed that the group membership probabilities had a conventional non-informative Dirichlet prior distribution: *G_i_* ~ Categorical(*prop_k_*) where *prop_k_* is the proportion of species in group *k* (for plants, *k* = 1, 2, 3). We used the gamma distribution to construct the Dirichlet prior: *g_k_* ~ Gamma(1,1); ${prop}_{k}={g_{k}}/{\sum_{k=1}^{K} g_{k}}$. See [Royle and Dorazio (2008)](#_ENREF_10) or [Kéry and Schaub (2012)](#_ENREF_7) for the detail explanation and usage of this distribution in hierarchical models. Based on the estimated group memberships of undetected species that were estimated to be present, we modelled their species-level parameters (and associated occurrence probabilities and frequencies for plants) with group-specific hyper-parameters.

1. Bayesian analysis by Markov chain Monte Carlo

We used conventional non-informative priors ([Royle & Dorazio 2008](#_ENREF_10)), e.g., Ω ~ Uniform(0,1); $\mu_{\beta_{0}}$, $\mu_{\beta_{1}}$ ~ Norm(0,1000); $\sigma_{\beta_{0}}$,$\sigma_{\beta_{1}}$ ~ Uniform(0,5). We ran three chains of 600,000 iterations for birds and plants, with different initial values, discarded the first 100,000 for birds, 300,000 for plants, and thinned each chain by 100. Markov chain convergence was examined using the $\hat{R}$ statistic (the Gelman-Rubin statistic), and $\hat{R}$for all the parameters was less than 1.1 indicating convergence ([Gelman & Hill 2007](#_ENREF_6)). Posterior distributions were summarized by their medians and 95% credible intervals (CIs).

1. Assessing the impact of DARs on SARs

It is not straightforward to test the effects of DARs on the form of SARs and abundance-area relationships even using the community-level hyper-parameters because we obtain community-level properties (e.g., species richness, total abundance) as derived parameters. Here we propose a way to test the effects of DARs by comparing SARs and abundance-area relationships derived from the developed procedures against those derived under constant density hypothesis (*β*_1_*_i_* = 1 for abundance and 0 for frequency models). That is, we construct community-level species richness and abundance under the constant density hypothesis (${\hat{R}cd}_{j}$ and ${\hat{N}cd}_{j}$, respectively), and we deal with hypothetical SARs and abundance-area relationships as predictions of null models. We first formulated expected values of ${\hat{R}cd}_{j}$ with an offset term in abundance models as follows:

$E[{\hat{R}cd}_{j}]=\sum_{i=1}^{s+m} \left\{ w_{i}\times\left[ 1-exp\left( -\exp\left( \hat{\beta}_{0i}+\log\left( A_{j} \right)+b_{ij} \right) \right) \right] \right\}$

eqn S15

where *s* and *m* is the number of observed and potential (augmented) species, respectively (this is essentially eqn S9 substituted for *λ* in eqn S8, but with *β*_1_*_i_ =* 1 and random site effects). We note that this equation should include the indicator variable of data augmentation *w_i_*, which is used to partition potential species into present or absent species. Expected value of ${\hat{N}cd}_{j}$ can be similarly formulated as follows (from eqn S9):

$E[{\hat{N}cd}_{j}]=\sum_{i=1}^{s+m} \left[ w_{i}\times\exp\left( \hat{\beta}_{0i}+\log\left( A_{j} \right)+b_{ij} \right) \right]$. eqn S16

In the case of the frequency model, we propose that expected values of ${\hat{R}cd}_{j}$ are composed of the linear predictor without slopes (i.e., eqn S11 substituted for $\psi$ in eqn S13, but with $\beta$_1_*_i_ =* ***β****_i_* = 0):

$E\left[ {\hat{R}cd}_{j} \right]=\sum_{i=1}^{s} \left\{ w_{i}\times\left[ 1-\left( 1-\left( 1/\left( 1+\exp\left( -\left( \hat{\beta}_{0i}+b_{ij} \right) \right) \right) \right) \right)^{{tp}_{j}} \right] \right\}$ eqn S17

as well as an expected frequency (based on a binomial distribution):

$E\left[ {\hat{N}cd}_{j} \right]=\sum_{i=1}^{s} \left\{ w_{i}\times\left[ {tp}_{j}\times\left( 1/\left( 1+\exp\left( -\left( \hat{\beta}_{0i}+b_{ij} \right) \right) \right) \right) \right] \right\}$.

eqn S18

These formulations indicate that we fixed mean values of *β*_1_*_i_* as 1 and 0 for birds and plants, respectively, and their standard deviations as 0. We treated their posterior distributions as predictions of null models. Because we intended to test the very effects of DARs, linear predictors included random site effects. In this respect, these null models may be called as neutral density models rather than constant density models.

References

Arrhenius, O. (1921) Species and area. *Journal of Ecology,* **9,** 95–99.

Barnagaud, J.-Y., Barbaro, L., Papaïx, J., Deconchat, M. & Brockerhoff, E.G. (2014) Habitat filtering by landscape and local forest composition in native and exotic New Zealand birds. *Ecology,* **95,** 78–87.

Chandler, R.B., King, D.I., Raudales, R., Trubey, R., Chandler, C. & Chávez, V.J.A. (2013) A small-scale land-sparing approach to conserving biological diversity in tropical agricultural landscapes. *Conservation Biology,* **27,** 785–795.

Chen, G., Kéry, M., Plattner, M., Ma, K. & Gardner, B. (2013) Imperfect detection is the rule rather than the exception in plant distribution studies. *Journal of Ecology,* **101,** 183–191.

Coleman, B.D., Mares, M.A., Willig, M.R. & Hsieh, Y.-H. (1982) Randomness, area, and species richness. *Ecology,* **63,** 1121–1133.

Gelman, A. & Hill, J. (2007) *Data analysis using regression and multilevel/hierarchical models*. Cambridge University Press, Cambridge.

Kéry, M. & Schaub, M. (2012) *Bayesian population analysis using WinBUGS: a hierarchical perspective*. Academic Press, San Diego.

McCullagh, P. & Nelder, J.A. (1989) *Generalized linear models,* Second edn. Chapman & Hall, London.

Otis, D.L., Burnham, K.P., White, G.C. & Anderson, D.R. (1978) Statistical-inference from capture data on closed animal populations. *Wildlife Monographs,* **62,** 3–135.

Royle, J.A. & Dorazio, R.M. (2008) *Hierarchical modeling and inference in ecology: the analysis of data from populations, metapopulations and communities*. Academic Press, Amsterdam.

Royle, J.A., Kéry, M., Gautier, R. & Schmid, H. (2007) Hierarchical spatial models of abundance and occurrence from imperfect survey data. *Ecological Monographs,* **77,** 465–481.

Yamaura, Y., Kéry, M. & Royle, J.A. (2016) Study of biological communities subject to imperfect detection: bias and precision of community *N*-mixture abundance models in small-sample situations. *Ecological Research,* **31,** 289-305.

Yamaura, Y., Royle, J.A., Kuboi, K., Tada, T., Ikeno, S. & Makino, S. (2011) Modelling community dynamics based on species-level abundance models from detection/nondetection data. *Journal of Applied Ecology,* **48,** 67–75.

Yamaura, Y., Royle, J.A., Shimada, N., Asanuma, S., Sato, T., Taki, H. & Makino, S. (2012) Biodiversity of man-made open habitats in an underused country: a class of multispecies abundance models for count data. *Biodiversity and Conservation,* **21,** 1365–1380.
