# Supplementary material for: Estimating species – area relationships by modeling abundance and frequency subject to incomplete sampling
Source: Ecol Evol. 2016 Jun 17;6(14):4836–48. doi: 10.1002/ece3.2244 (PMC4979711; doi:10.1002/ece3.2244)
Supplement: Supplementary file 2 — Appendix S2. Study area and details of sampling sites. [file ECE3-6-4836-s002.docx]

**Appendix S2. Study area and details of sampling sites.**


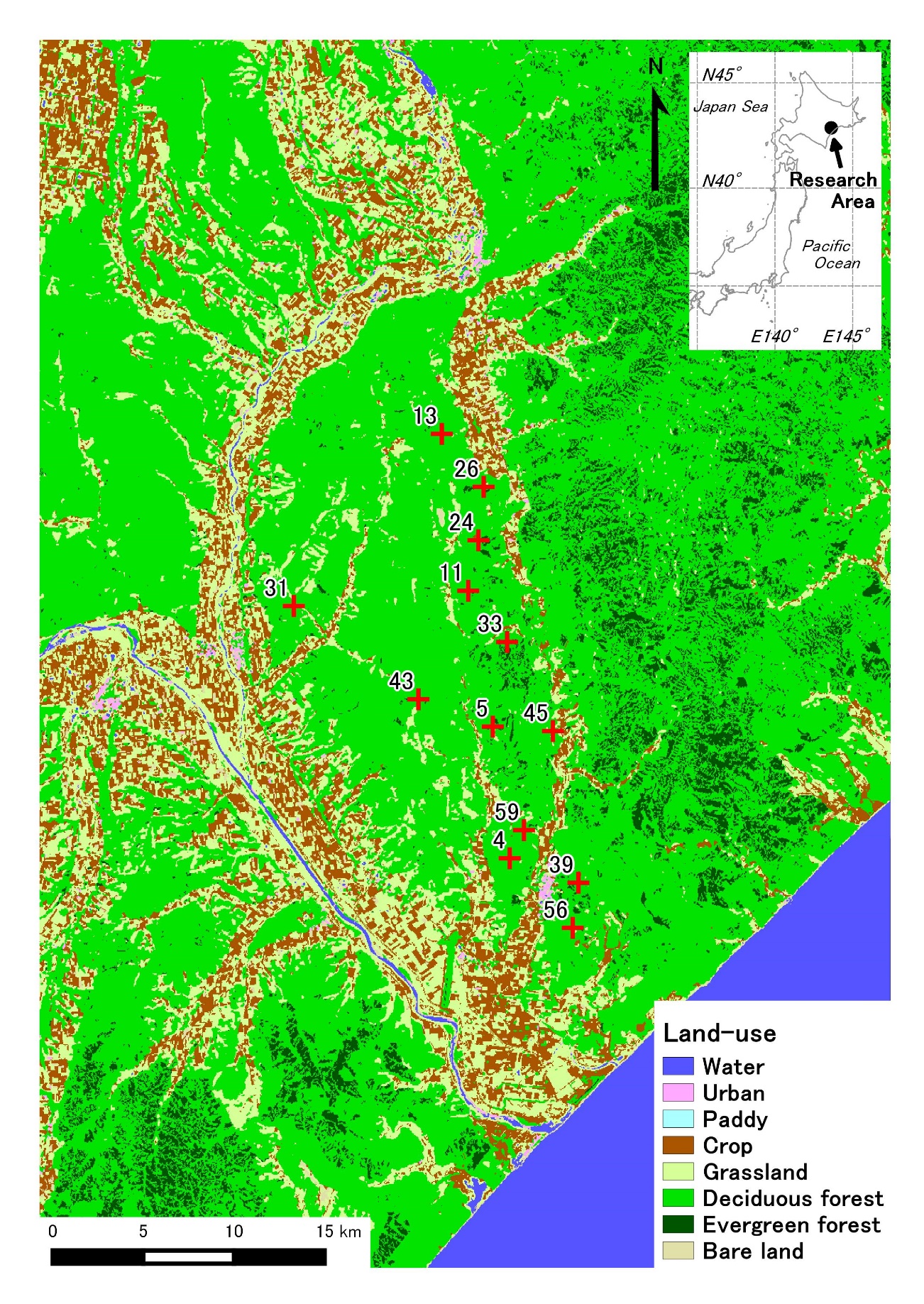


Fig. 1. Study area and location of sampling sites. Please note that larch plantation is included in deciduous forest because larch is a deciduous conifer tree. Sampled patches are shown as crosses with their ID numbers.

Base land-use map is the research product datasets of "Daichi" (AVNIR-2 of ALOS) processed in EORC, JAXA (http://www.eorc.jaxa.jp/ALOS/lulc/lulc_jindex.htm). © JAXA.

Table 1. Details of sampling sites.

| ID^a^ | ID^b^ | Stand age  (in 2011) | Area (ha) | Area for  plant sampling (ha)^c^ | Area class | Number of  plot^d^ | Adjacent patch |
| --- | --- | --- | --- | --- | --- | --- | --- |
| 4 | 1 | 4 | 2.7 | - | Small | 35 |  |
| 5 | 2 | 4 | 9.9 | - | Large | 154 | Three patches, totally 11.27 ha |
| 11 | 3 | 4 | 5.9 | - | Medium | 93 |  |
| 13 | 4 | 5 | 1.6 | - | Small | 20 |  |
| 24 | 5 | 4 | 4.8 | - | Medium | 66 | One 10.7 ha patch |
| 26 | 6 | 5 | 6.2 | - | Large | 90 |  |
| 31 | 7 | 5 | 5.1 | - | Medium | 73 |  |
| 33 | 8 | 4 | 2.2 | 1.9 | Small | 29 | One 0.22 ha patch |
| 39 | 9 | 4 | 6.4 | 4.8 | Large | 72 |  |
| 43 | 10 | 5 | 3.8 | - | Medium | 55 |  |
| 45 | 11 | 5 | 1.7 | 1.5 | Small | 26 |  |
| 56 | 12 | 6 | 10.0 | - | Large | 161 |  |
| 59 | 13 | 6 | 1.3 | - | Small | 20 | One 0.1 ha patch |

^a^Site identity used in the map. ^b^Site identity used in the analysis. ^c^Area used for sampling of plant plots. ^d^Number of plant plots.
