# Supplementary material for: Estimating species – area relationships by modeling abundance and frequency subject to incomplete sampling
Source: Ecol Evol. 2016 Jun 17;6(14):4836–48. doi: 10.1002/ece3.2244 (PMC4979711; doi:10.1002/ece3.2244)
Supplement: Supplementary file 3 — Appendix S3. Simulation experiments on the roles of density‐area relationships on species‐area relationships [file ECE3-6-4836-s003.docx]

**Appendix S3. Simulation experiments on the roles of density-area relationships on species-area relationships.**

Methods

We expect that DARs for the set of species comprising a community (the values of *β*_1_*_i_* in the linear predictors of species-level models) will affect the shape of the community-level SARs. To test this expectation, we conducted simulations to examine the effect of varying the individual species DARs on the behavior of the community-level SARs and the community-level abundance/frequency-area relationships. We used the two models described above: multispecies abundance and frequency models with only *β*_0_*_i_* and *β*_1_*_i_*. In the simulations, we used communities of 50 and 400 species and patch areas of 0.01 to 30 ha and 0.0001 (single plot size) to 30 ha for the abundance and frequency models, respectively. We assumed that plot size for the plant survey was 1 × 1 m^2^. As is common in hierarchical community occupancy ([Royle & Dorazio 2008](#_ENREF_1)) and abundance ([Yamaura *et al.* 2011](#_ENREF_2); [Yamaura *et al.* 2012](#_ENREF_3)) models, we assumed that the species-specific effect of area on mean abundance (or frequency) varies among species so that *β*_0_*_i_* and *β*_1_*_i_* in both models are generated from normal distributions:

***β****_i_* ~ Normal(***μ***,***σ*^2^**)

where ***μ*** is the mean of ***β****_i_* across all the species, and ***σ*^2^** are the variances. We chose values of $\mu_{\beta_{0}}$ and $\sigma_{\beta_{0}}^{2}$ motivated by the results of fitting the abundance and frequency models to bird and plant data from *Larix leptolepis* plantation patches. However, we varied $\mu_{\beta_{1}}$ and $\sigma_{\beta_{1}}^{2}$ to create four scenarios in which on-average individual species DARs either implied that density (or occurrence probability) was or was not a function of patch area (Table 1). We generated abundance or frequency of each species at each patch under each scenario for 100 replicates, and estimated community-level abundance or frequency and species richness at each patch and for each replicate. We also calculated expected species richness across patches using eqns S8 and S13 from Appendix S1 with the simulated values of *β*_0_*_i_* and *β*_1_*_i_*.

Results

For the simulations of the abundance model, as expected, results showed that the dependence of individual species densities on patch area affects the responses of community-level abundance and species richness to patch area (Fig. 1). This is because area-dependency of the probability that at least one individual occurs and the probability that a species occurs on at least one plot (here we call occupancy probability) greatly differ among the scenarios (Fig. 2).

Community-level abundance greatly differed among the four scenarios, and simulations in which the average DARs had *β*_1_ >1 or *β*_1_ <1 increased or decreased community-level abundance, respectively, relative to control scenarios with population densities independent of patch area (*β*_1_ =1). DARs with *β*_1_ >1 or *β*_1_ <1 made the shape of SARs steeper and shallower (viz. increase or decrease the log-log slope), respectively, relative to control scenarios. Although the simulation results for the multispecies frequency model were similar to those of the abundance model, one exception was that species richness decreased in larger patches when *β*_1_ <0 creating a somewhat humped SAR (Figs. 1-2).

Table 1. Means and standard deviations of *β*_1_*_i_* in four scenarios in multispecies abundance and frequency models. We set mean and SD of *β*_0_*_i_* as –2.13 and 1.5 for abundance model, –7.83 and 3.35 for frequency model, respectively, throughout the experiments.

| Scenario | Mean | SD |
| --- | --- | --- |
| Abundance model |  |  |
| 1. Density independent of patch area* | 1 | 0 |
| 2. Density independent of patch area with species variation | 1 | 0.33 |
| 3. Density higher in small patches with species variation | 0.7 | 0.33 |
| 4. Density higher in large patches with species variation | 1.5 | 0.33 |
|  |  |  |
| Frequency model |  |  |
| 1. Occurrence probability (OP) independent of patch area* | 0 | 0 |
| 2. OP independent of patch area with species variation | 0 | 0.08 |
| 3. OP higher in small patches with species variation | –0.1 | 0.08 |
| 4. OP higher in large patches with species variation | 0.1 | 0.08 |

* Control scenario in which density or occurrence probability is not a function of patch area.

Fig. 1. Simulated community-level state variables in relation to patch area for a community with a maximum of (a, b) 50 species under the abundance model and (c, d) 400 species under the frequency model. Among 100 replicates, we plotted the median values against the patch area. (a, c) Species richness as a function of area (SAR) under three scenarios (see Table 1 for details). SARs for scenario 1 are omitted because these curves were almost identical to those of scenario 2. Expected values derived from eqns S8 and S13 from Appendix S1 are also shown for scenario 2 (depicted lines are those with the median values at the largest patch area). (b, d) Community-level abundance and frequency as a function of area under the four scenarios.

Fig. 2. Predicted species richness and occupancy probability of individual species for abundance (a,b) and frequency (c, d) models. Each figure is the result of single simulation of four scenarios: (a) negative DAR, (b) positive DAR, (c) negative occurrence probability-area relationship, and (d) positive occurrence probability-area relationship. Grey lines show occupancy probabilities of individual species, while black lines show expected species richness, which are the summed occupancy probabilities of individual species.

References

Royle, J.A. & Dorazio, R.M. (2008) *Hierarchical modeling and inference in ecology: the analysis of data from populations, metapopulations and communities*. Academic Press, Amsterdam.

Yamaura, Y., Royle, J.A., Kuboi, K., Tada, T., Ikeno, S. & Makino, S. (2011) Modelling community dynamics based on species-level abundance models from detection/nondetection data. *Journal of Applied Ecology,* **48,** 67-75.

Yamaura, Y., Royle, J.A., Shimada, N., Asanuma, S., Sato, T., Taki, H. & Makino, S. (2012) Biodiversity of man-made open habitats in an underused country: a class of multispecies abundance models for count data. *Biodiversity and Conservation,* **21,** 1365-1380.
