# Supplementary material for: Estimating species – area relationships by modeling abundance and frequency subject to incomplete sampling
Source: Ecol Evol. 2016 Jun 17;6(14):4836–48. doi: 10.1002/ece3.2244 (PMC4979711; doi:10.1002/ece3.2244)
Supplement: Supplementary file 4 — Appendix S4. Comparison between multispecies models and traditional estimators using field data and simulation experiments. [file ECE3-6-4836-s004.docx]

**Appendix S4. Comparison between multispecies models and traditional estimators using field data and simulation experiments.**

Introduction

Because the comparisons between traditional species richness estimators and hierarchical models are limited ([Iknayan *et al.* 2014](#_ENREF_9)), differences in species richness estimates between these two approaches remains unclear. Therefore, as a first step, we compared species richness estimates in individual patches to those of traditional nonparametric estimators. We also conducted simulation experiments to examine the bias of species richness estimators for the models we develop and for traditional estimators subject to incomplete sampling.

Methods

1. Comparison using the field data

To compare the species richness estimates between multispecies models and traditional estimators, we estimated species richness in each patch based on the detected abundances and observed frequencies for birds and plants, respectively. We applied abundance-based estimators (Chao and ACE) for birds and incidence-based estimators (Chao, first and second order Jackknife, and bootstrap) for plants using ‘estimateR’ and ‘specpool’ functions in ‘vegan’ R package ver. 2.2-1 ([Oksanen *et al.* 2015](#_ENREF_12)). For birds, we also obtained the estimates of species richness using the linear regressions with observed species richness as a response variable, and logarithmically transformed patch area (ha) as an explanatory variable. For plants, we also constructed the regression models with the total area of sampling plots (ha) as an explanatory variable, and extrapolated the models to obtain patch-level estimates using the patch area as an explanatory variable.

1. Simulation experiments

We conducted simulation experiments to examine the bias of species richness estimators subject to incomplete sampling given that true species richness is known. Species pool size was 40, and they shared 0.5 mean density ($\mu_{\beta_{0}}$= log[0.5]) and 0.5 detection probability ($\text{μ}_{r}$= log[1/[1/0.5 – 1]] = 0). We used $\sigma_{\beta_{0}}$= 1.5 and $\sigma_{r}$= 0.5, which was motivated by the analysis of our bird data set. This means that 40 individual species had varied density and detection probability, but means were set to 0.5. We also conducted the experiments with 0.9 mean detection probability (other settings were the same). For simplicity, we assumed that there were five sites with the same area and no covariates; therefore, each species had the same expected abundance across five sites: log(*λ_i_*_1,2,3,4,5_) = *β_i_*_0_ + log(*A*). Abundance of each species and site (*z_ij_*) was assumed to follow a Poisson distribution: *z_ij_* ~ Poisson(*λ_i_*). Each site was partially covered by the sampling plot, *a* <*A*; *φ = a* / *A*. Individuals were exposed to the sampling (*N_ij_*) following a binomial process: *N_ij_* ~ Binomial(*z_ij_*,*φ*). We then conducted a virtual survey by counting the number of detected individuals (*y_ijv_*) assuming a binomial process: *y_ijv_* ~ Binomial(*N_ij_*,*p_i_*). A virtual surveyor visited each site five times (*v* = 5).

We applied our multispecies abundance model based on the same assumptions used in the data generation processes to *y_ijv_* given *a* and *A*. We used 80 augmented species to account for the existence of undetected species, and estimated site-specific species richness for each of five sites via jagsUI R package ver. 1.3.1 ([Kellner 2015](#_ENREF_10)). We ran three chains of 110,000 iterations, discarded the first 10,000, and did not thin the chains. Model convergence was assumed when $\hat{R}$ statistic of site-specific species richness was less than 1.1; otherwise, we ran one or multiple sets of additional sets of 110,000 iterations until we achieved chain convergence, using the function ‘autojags’ of jagsUI. We also estimated site-specific species richness using abundance-based estimators (Chao and ACE) using the maximum number of detected individuals during the five visits (max[*y_ij_*_1-5_]). We then obtained the bias of species richness estimates for each site by subtracting corresponding true values from the estimates, and obtained mean values of the biases across five sites.

We constructed four scenarios to examine the effects of area of interest and spatial coverage of sampling plots: small area (1.1 ha) with small (0.11 ha) and large coverage (1 ha); large area (10 ha) with small (1 ha) and large coverage (9 ha). We replicated the data generation and model fitting process ten times for each scenario, and obtained ten mean values of biases for each species richness estimators. We used 80,000 burn-in and 180,000 iterations for the large area and large coverage scenario.

Results

1. Comparison using the field data

For birds, traditional species richness estimators generated estimates higher than those of the multispecies model, and sometimes estimates were more than twice those from the multispecies model (Fig. 1). For plants, in contrast to the results for birds, estimates of traditional species richness estimators were substantially lower than those of multispecies models (Fig. 2). For example, traditional estimates were only 1.3-1.5 times higher than observed species richness for the whole community data, while estimates from the multispecies models were 4.4 times higher. However, if we fit regression models of observed species richness with the total area of sample plots in each patch as the predictor variable, and extrapolate these curves to generate predicted species richness for patch areas the estimates of species richness increased towards the estimates of multispecies models (blue line in Fig. 2).

1. Simulation experiments

Traditional species richness estimators were strongly affected by the spatial coverage of sampling. Estimates in both small and large areas with low coverage produced underestimates, while those in the small area with high coverage led to overestimates (Fig. 3). In the small area with low coverage scenario, mean estimates of ACE were not available since in seven of the ten replications all species had less than two detected individuals in one of the five sites. The large area-high coverage scenario had the lowest bias (mean biases of Chao and ACE were 4.2 and 4.5, respectively), and these values decreased to 2.4, 3.0, respectively, when mean detection probability was increased to 0.9 (Fig. 4); but other general patterns and the magnitude of biases were not greatly changed. Species richness estimates for multispecies models were unbiased, but the uncertainty was higher with low coverage (Figs. 3-4).

Discussion

Abundances of most bird species in small patches are expected to be low. Because traditional species richness estimators are based on the number of species with only one or two individuals ([singletons and doubletons, respectively: Gotelli & Colwell 2001](#_ENREF_8)), their estimates were higher than those from multispecies models, and unrealistically high in some patches (Fig. 1). This observation was confirmed in simulation experiments in which true species richness was known. In the small area-high coverage scenario, traditional estimators overestimated while multispecies models generated unbiased estimates (Figs. 3-4). On the other hand, plant species richness estimates from traditional estimators were substantially lower than those of multispecies models (Fig. 2). But we expect that 400 additional species could occur in these habitats. Underestimation by traditional estimators subject to low spatial coverage was also confirmed by the simulation experiments irrespective of area (Figs. 3-4). However, the unbiased estimates from our multispecies models are not unexpected because the same assumptions used in the models were used in the data generation. Nevertheless, these results suggest that traditional estimators do not work well with low spatial coverage. Brose, Martinez & Williams ([2003](#_ENREF_2)) and [O'Hara (2005)](#_ENREF_11) also showed similar problems with traditional estimators, and suggested the need to develop better estimators that also account for heterogeneous detection probabilities. The models we developed are candidates to overcome these problems, and can account for habitat heterogeneity using additional covariates. The problem of underestimation was only partly resolved by extrapolation of plot-based regression models to predict patch area species richness, since the estimates were still underestimates and their uncertainties would be high.

Because traditional estimators are suggested to make lower bound estimates of species pool size ([Chao *et al.* 2009](#_ENREF_3)), condition-dependent biases of traditional estimators in this study may also be reasonable. Traditional estimators are non-parametric methods, and do not formulate imperfect detection and incomplete spatial coverage, and do not distinguish between these processes. Although we made the minimal assumptions in our models, and used random effects to consider the un-modeled variations in the field data, our proposed framework also has limitations as the data augmentation did not work for birds. Recently, traditional estimators have been actively developed to consider abundance information ([Chao *et al.* 2014](#_ENREF_4); [Chao *et al.* 2015](#_ENREF_5)), un-sampled areas ([Chao & Lin 2012](#_ENREF_6)), and relevant covariates ([Böhning *et al.* 2013](#_ENREF_1)). Interestingly, [Colwell *et al.* (2012)](#_ENREF_7) proposed the use of Poisson and binomial distributions to interpolate rarefaction curves for abundance and frequency data, respectively. Some of the model structure are shared with our models, although without distinguishing formally between observation and process models.

Fig. 1. Species richness of early-successional birds in larch plantation patches. Solid and dotted black lines indicate the median and 95% CIs derived from multispecies abundance model (HM), respectively. Vertical line indicates the smallest area of our sampled patches. Estimated values smaller than this area are derived from extrapolation of the model. Solid and dotted grey lines were predictions from null models under constant density hypothesis. Red line indicates predictions from the regression models (parameter estimates were shown in Table 1).

Fig. 2. Plant species richness in relation to area of larch patches for (a) the entire plant community, (b) early successional species, (c) mature forest species, and (d) exotic species. Estimated values were derived from the multispecies frequency model. See Fig. 1 for detailed descriptions of symbols. Four figures have different ranges of vertical axes. Although we encountered 314 species throughout the survey, we only observed a subset of these species in each patch because of the field survey did not cover the entire area of each patch (incomplete spatial coverage). Hence, our estimated values for species richness which account for incomplete spatial coverage are substantially higher than the observed values of species richness. Red lines indicate predictions from the regression models with patch area. Blue lines indicate predictions from the extrapolation of regression models with plot area; after the model construction, we predicted the patch-level estimates using patch area as an explanatory variable (parameter estimates were shown in Table 1).

Table 1. Parameter estimates of regression models for observed species richness.

|  | Patch area model | | | | |  | Plot area model (only for plants) | | | | |
| --- | --- | --- | --- | --- | --- | --- | --- | --- | --- | --- | --- |
|  | Estimate | SE | t-value | p-value | R^2^ |  | Estimate | SE | t-value | p-value | R^2^ |
| (a) Bird species |  |  |  |  |  |  |  |  |  |  |  |
| Intercept | 2.58 | 0.49 | 5.27 | 0.00 | 0.84 |  |  |  |  |  |  |
| log(area) | 2.46 | 0.33 | 7.58 | 0.00 |  |  |  |  |  |  |  |
|  |  |  |  |  |  |  |  |  |  |  |  |
| (b) All plant species | |  |  |  |  |  |  |  |  |  |  |
| (Intercept) | 64.83 | 9.98 | 6.49 | 0.00 | 0.76 |  | 319.40 | 27.86 | 11.46 | 0.00 | 0.83 |
| log(area) | 38.64 | 6.62 | 5.84 | 0.00 |  |  | 38.85 | 5.31 | 7.32 | 0.00 |  |
|  |  |  |  |  |  |  |  |  |  |  |  |
| (c) Plant early-successional species | | |  |  |  |  |  |  |  |  |  |
| (Intercept) | 22.99 | 4.23 | 5.43 | 0.00 | 0.73 |  | 124.31 | 12.31 | 10.10 | 0.00 | 0.80 |
| log(area) | 15.44 | 2.81 | 5.50 | 0.00 |  |  | 15.45 | 2.35 | 6.59 | 0.00 |  |
|  |  |  |  |  |  |  |  |  |  |  |  |
| (d) Plant mature forest species | | |  |  |  |  |  |  |  |  |  |
| (Intercept) | 39.16 | 7.46 | 5.25 | 0.00 | 0.59 |  | 170.32 | 22.31 | 7.64 | 0.00 | 0.67 |
| log(area) | 19.62 | 4.95 | 3.97 | 0.00 |  |  | 20.09 | 4.25 | 4.73 | 0.00 |  |
|  |  |  |  |  |  |  |  |  |  |  |  |
| (e) Plant exotic species | |  |  |  |  |  |  |  |  |  |  |
| (Intercept) | 2.68 | 0.98 | 2.72 | 0.02 | 0.73 |  | 24.77 | 3.61 | 6.87 | 0.00 | 0.68 |
| log(area) | 3.59 | 0.65 | 5.49 | 0.00 |  |  | 3.31 | 0.69 | 4.82 | 0.00 |  |

Fig. 3. Boxplots of the mean biases of three species richness estimators across five sites. Four scenarios with different area of concern and spatial coverage of sampling plots were separately depicted, and ten replications were conducted for each scenario. MSAM means multispecies abundance model, and horizontal grey lines indicate no bias of the estimates. Mean individual-level detection probability across species was set at 0.5.

Fig. 4. Boxplots of the mean biases of estimates derived from three species richness estimators across five sites in simulation experiments.

Four scenarios with different area and spatial coverage of sampling plots were separately depicted, and ten replications were conducted for each scenario. MSAM means multispecies abundance model, and horizontal grey lines indicate no bias of the estimates. Mean individual-level detection probability across species was set at 0.9. In the small area with low coverage scenario, mean estimates of ACE were not available in seven of ten replications due to the computational reason.

References

Böhning, D., Vidal-Diez, A., Lerdsuwansri, R., Viwatwongkasem, C. & Arnold, M. (2013) A generalization of Chao's estimator for covariate information. *Biometrics,* **69,** 1033–1042.

Brose, U., Martinez, N.D. & Williams, R.J. (2003) Estimating species richness: sensitivity to sample coverage and insensitivity to spatial patterns. *Ecology,* **84,** 2364–2377.

Chao, A., Colwell, R.K., Lin, C.-W. & Gotelli, N.J. (2009) Sufficient sampling for asymptotic minimum species richness estimators. *Ecology,* **90,** 1125–1133.

Chao, A., Gotelli, N.J., Hsieh, T.C., Sander, E.L., Ma, K.H., Colwell, R.K. & Ellison, A.M. (2014) Rarefaction and extrapolation with Hill numbers: a framework for sampling and estimation in species diversity studies. *Ecological Monographs,* **84,** 45–67.

Chao, A., Hsieh, T.C., Chazdon, R.L., Colwell, R.K. & Gotelli, N.J. (2015) Unveiling the species-rank abundance distribution by generalizing the Good-Turing sample coverage theory. *Ecology,* **96,** 1189–1201.

Chao, A. & Lin, C.-W. (2012) Nonparametric lower bounds for species richness and shared species richness under sampling without replacement. *Biometrics,* **68,** 912–921.

Colwell, R.K., Chao, A., Gotelli, N.J., Lin, S.-Y., Mao, C.X., Chazdon, R.L. & Longino, J.T. (2012) Models and estimators linking individual-based and sample-based rarefaction, extrapolation and comparison of assemblages. *Journal of Plant Ecology,* **5,** 3–21.

Gotelli, N.J. & Colwell, R.K. (2001) Quantifying biodiversity: procedures and pitfalls in the measurement and comparison of species richness. *Ecology Letters,* **4,** 379–391.

Iknayan, K.J., Tingley, M.W., Furnas, B.J. & Beissinger, S.R. (2014) Detecting diversity: emerging methods to estimate species diversity. *Trends in Ecology & Evolution,* **29,** 97–106.

Kellner, K. (2015) jagsUI: a wrapper around rjags to streamline JAGS analyses.

O'Hara, R.B. (2005) Species richness estimators: how many species can dance on the head of a pin? *Journal of Animal Ecology,* **74,** 375–386.

Oksanen, J., Blanchet, F.G., Kindt, R., Legendre, P., Minchin, P.R., O'Hara, R.B., Simpson, G.L., Solymos, P., Stevens, M.H.H. & Wagner, H. (2015) vegan: community ecology R package. <http://CRAN.R–project.org/package=vegan>.
